# MicroRNA-21 expression is associated with overall survival in patients with glioma

**DOI:** 10.1186/1746-1596-8-200

**Published:** 2013-12-10

**Authors:** Lin Wu, Gang Li, Dayun Feng, Huaizhou Qin, Li Gong, Jian Zhang, Zhiguo Zhang

**Affiliations:** 1 Department of Biochemistry and Molecular Biology, State Key Laboratory of Cancer Biology, the Fourth Military Medical University, Xi’an, China; 2 Department of Neurosurgery and Institute for Functional Brain Disorders, Tangdu Hospital, the Fourth Military Medical University, Xi’an, China; 3 Department of Pathology, Tangdu Hospital, the Fourth Military Medical University, Xi’an, China; 4 Postdoctoral research station of Neurosurgery, Wuhan General Hospital of Guangzhou Command, PLA, Wuhan, China

**Keywords:** Glioma, MiR-21, Overall survival

## Abstract

**Background:**

MicroRNA-21 has been proved to be associated with glioma proliferation and invasion; thus, we sought to clarify the clinical value of miR-21 expression in glioma tissues with WHO grade I to IV.

**Methods:**

One hundred and fifty-two pairs of human gliomas and non-neoplastic brain tissues were evaluated using real-time PCR. The association of miR-21 expression with clinicopathological factors or the prognosis of glioma patients was also analyzed. In this study, survival analysis was performed using the Kaplan-Meier method and Cox’s proportional hazards model.

**Results:**

MiR-21 was more greatly expressed in glioma tissues compared to the corresponding non-neoplastic brain tissues (P < 0.001). This observed high miR-21 expression was significantly associated with high pathological grades and the Karnofsky performance score of glioma patients. In addition, overall patient survival for those with low miR-21 expression was significantly longer than those patients with high miR-21 expression (P < 0.001). Moreover, multivariate Cox regression analysis indicated that miR-21 might be an independent prognostic marker for glioma patient survival.

**Conclusions:**

Our data show that miR-21 may be a candidate independent marker for gliomas, especially those with high pathological grades, and this could also be a potential therapeutic target for molecular glioma therapy.

**Virtual slide:**

The virtual slide(s) for this article can be found here: http://www.diagnosticpathology.diagnomx.eu/vs/1445749171109834.

## Background

Glioma, the most common primary malignant brain tumor arising from the brain orspinal cord tissue, has a tendency to invade the surrounding brain tissue. Gliomas represent approximately one-third of all intrinsic neoplasms of the central nervous system in both adults and children. The World Health Organization (WHO) classification divides gliomas into grades I through IV, and these values correspond to increasing levels of malignancy, including well-differentiated low grade, astrocytomas and glioblastoma multiforme [1]. Glioma has a poor prognosis due to the characteristic progressive overgrowth and diffuse invasion. Although the WHO classification can serve as a criterion to predict the patient clinical outcomes, several recent studies have indicated that this criteria alone may not be sufficient to estimate patient prognosis [2,3]. Therefore, investigating molecular mechanisms of gliomas may produce better prognostic markers to anticipate patient survival.

MicroRNAs (miRNAs) are a class of small, evolutionarily conserved, short non-coding endogenous RNA molecules that regulate gene expression at the post-transcriptional level [4]. Primary transcripts of miRNAs (pri-miRNA) are generated by RNA polymerase II and then they are sequentially processed by RNase III enzymes, Drosha and Dicer, to first produce pre-miRNAs and finally mature miRNAs. MiRNAs act by base-pairing with their target mRNAs according to the degree of complementarities with their target 3′ untranslated regions (UTRs) leading to their translational regulation and/or direct cleavage [5,6]. MiRNAs have been demonstrated to play normal physiologic roles in cell proliferation and differentiation, epithelial-mesenchymal transition, apoptosis and metabolism [7,8]. In addition, studies suggest that miRNAs also play important roles in tumorigenesis and tumor progression, acting as oncogenes or tumor suppressors depending on their target genes. Finally, some miRNAs may be markers for cancer diagnosis and prognosis [9,10].

In the present study, we focus on miR-21, which has been demonstrated to act as either an oncogenic miRNA or an anti-oncomiR (miRNA that negatively regulates oncogenes) in various human malignancies including glioma [11-15]. However, to our knowledge, no correlation between miR-21 and patient prognosis has not been addressed in glioma at this time. To investigate this problem, miR-21 expression in human gliomas and nonneoplastic brain tissues was measured using real-time quantitative RT-PCR assay. The association of miR-21 with clinicopathological factors or glioma patient prognosis was also statistically analyzed.

## Methods and materials

### Patients and tissue samples

This study was approved by the Research Ethics Committee of Tangdu Hospital of Fourth Military Medical University, P. R. China. Written informed consent for the following pathological analysis or experiments was obtained from all of the patients before his/her surgery. All specimens were handled and made anonymous according to the ethical and legal standards.

One hundred and fifty-two pairs of glioma tissues including adjacent non-neoplastic brain tissues were selected, and the pathological information was retrieved from the archives of the Pathology Department of Tangdu Hospital, Fourth Military Medical University, P. R. China, from 2000 and 2009. None of the patients had received chemotherapy or radiotherapy prior to surgery. All the samples were resected from primary surgery, and the tumor tissues and the adjacent non-neoplastic brain tissues were divided by tissue laser microdissection [16]. The specimens were snap-frozen in liquid nitrogen and stored at -80°C for real-time PCR assay. The clinicopathological features and the treatment strategies of all the patients were indicated in Table 1.

**Table 1:** The clinicopathological features and the treatment strategies of all 152 patients with gliomas

**Features**	**WHO I**	**WHO II**	**WHO III**	**WHO IV**
**No. of cases**	31	30	32	59
**Mean age (Year)**	44.1	45.9	42.9	46.5
**Gender**				
**Male**	18	14	17	29
**Female**	13	16	15	30
**KPS**				
**≥70**	29	20	10	3
**<70**	2	10	22	56
**Surgery**				
**Total resection**	31	30	22	32
**Partial resection**	0	0	10	27
**Adjuvant treatment**				
**Radiotherapy**	0	0	23	29
**Chemotherapy**	0	2	4	1
**Radiotherapy + chemotherapy**	0	0	4	29

### Isolation of total RNA and real-time PCR analysis

MiR-21 expression in glioma and adjacent nonneoplastic brain tissues was measured by real-time quantitative RT-PCR. Total RNA was isolated from frozen samples using Trizol reagent (Invitrogen, CA, U.S.A.) according to the manufacturer’s protocol. The TaqMan microRNA assay and TaqMan universal PCR master mix were used to detect the expression of miRNA-21, and the *U6* gene was used as an internal control to normalize variances. Relative quantification of target miRNA expression was evaluated using the comparative cycle threshold (CT) method. Each sample was examined in triplicate and the raw data were presented as the relative quantity of target miRNA, normalized with respect to *U6*.

### Statistical analysis

Statistical analysis was performed using the SPSS 13.0 for Windows (SPSS Inc, IL, USA). The associations between miR-21 expression and clinical characteristics were evaluated by Mann-Whitney U test. Survival curves were estimated by the Kaplan-Meier method, and data were analyzed with the log-rank test. Cox proportional hazards analysis was performed to calculate the hazard ratio (HR) and the 95% confidence interval (CI) to evaluate the association between miR-21 expression and survival. In addition, a multivariate Cox regression was performed to adjust for other covariates. A value of P < 0.05 was considered statistically significant.

## Results

### Characteristics of patients

The characteristics of the 152 glioma patients involved in the study cohort are shown in Table 1. Of the 152 glioma patients, 74 were female (78 were male). The mean patient age was 45.1 years (range: 5-80 years-of-age). According to the WHO classification, 31, 30, 32, and 59 of the 152 glioma patients were classified as grade I, II, III, and IV, respectively. The Karnofsky performance score (KPS) to assess the well-being of 62 glioma patients was higher than 70 (able to care for self but unable to do active work), and the KPS of 90 patients was lower than 70.

### Increased expression of miR-21 in glioma tissues

**Figure 1:**
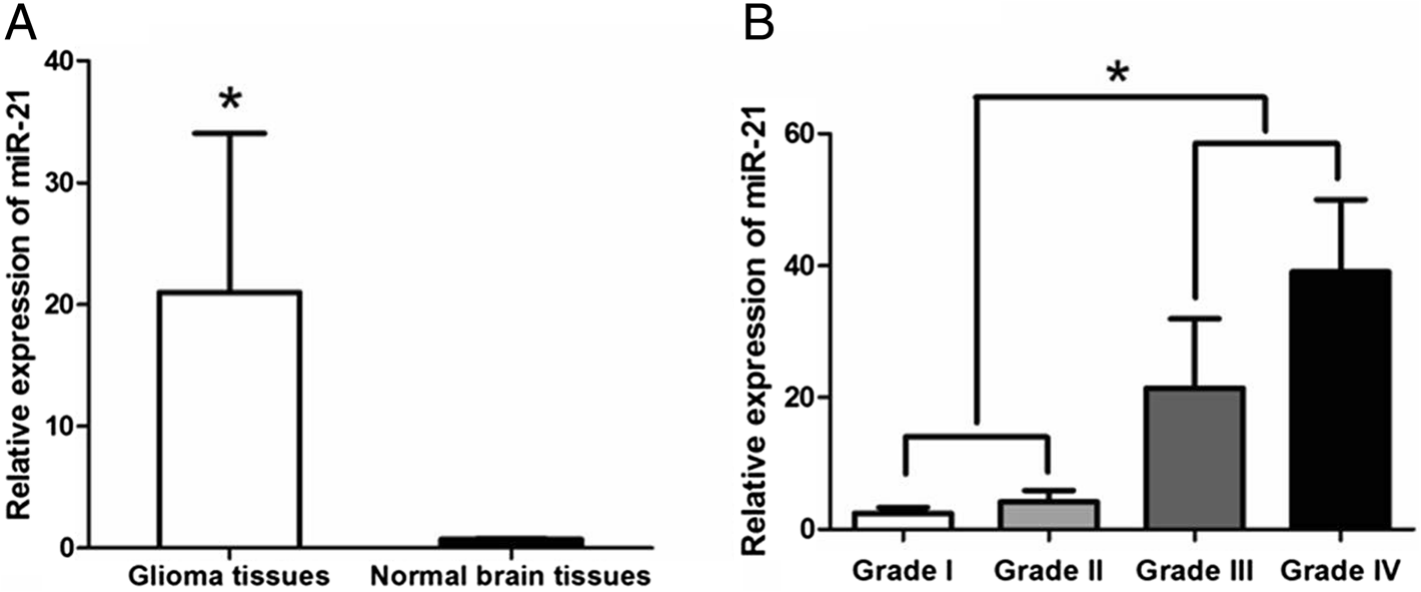
**The expression levels of miR-21 in 152 pairs of glioma and adjacent non-neoplatic brain tissues detected by Real-time PCR analysis. (A)** Expression levels of miR-21 in glioma and non-neoplastic brain tissues respectively. **(B)** Expression levels of miR-21 in 152 glioma tissues with pathological grades I to IV and homologous non-neoplastic brain tissues.

MiR-21 expression was detected in 152 pairs of glioma and adjacent non-neoplastic brain tissues. Normalized to the *U6* gene, the relative miR-21 expression in glioma samples was 20.99 ± 13.04 (mean ± SD), whereas the relative miR-21 expression detected in adjacent non-neoplastic normal tissues was 0.73 ± 0.05 (Figure 1A). We also observed that miR-21 expression in high-grade (III-IV) gliomas was higher than that observed in low-grade (I-II) gliomas (P < 0.001) (Figure 1B) and this increase was statistically significant.

### Association between miR-21 expression and clinicopathological characteristics of gliomas

The correlation of miR-21 expression with different clinicopathological parameters in gliomas was illustrated in Table 2. A close correlation was observed between miR-21 expression and the WHO glioma pathological grade (P < 0.001). Based on these data, the median miR-21 expression in all 152 glioma tissues was 20.99, thus we classified all glioma samples into 2 groups: low expression (mean expression value 3.25, n = 61), and high expression groups (mean expression value 32.87, n = 91). A significant relationship was also observed between miR-21 expression and the KPS (P < 0.001). However, there was no significant association between miR-21 expression and gender or age at diagnosis (Table 2).

**Table 2:** Association of miR-21 expression level in glioma tissues with gender, age, Karnofsky performance score (KPS) and World Health Organization (WHO) grade

**Clinicopathological features**	**No. of cases**	**miR-21 expression**		***P***
		**Low (n, %)**	**High (n,%)**	
**WHO Grade**				<0.001
**I**	31	31 (100%)	0 (0%)	
**II**	30	30 (100%)	0 (0%)	
**III**	32	18 (56.3%)	14 (43.7%)	
**IV**	59	3 (5%)	56 (95%)	
**Gender**				0.34
**Male**	78	45 (57.7%)	33 (42.3%)	
**Female**	74	37 (50%)	37 (50%)	
**Age**				0.23
**<50**	91	49 (53.8%)	42 (46.2%)	
**≥50**	61	33 (54.1%)	28 (45.9%)	
**KPS**				<0.001
**≥70**	62	57 (91.9%)	5 (8.1%)	
**<70**	90	25 (27.8%)	65 (72.2%)	

### Relationship of miR-21 expression with overall glioma patient survival

**Figure 2:**
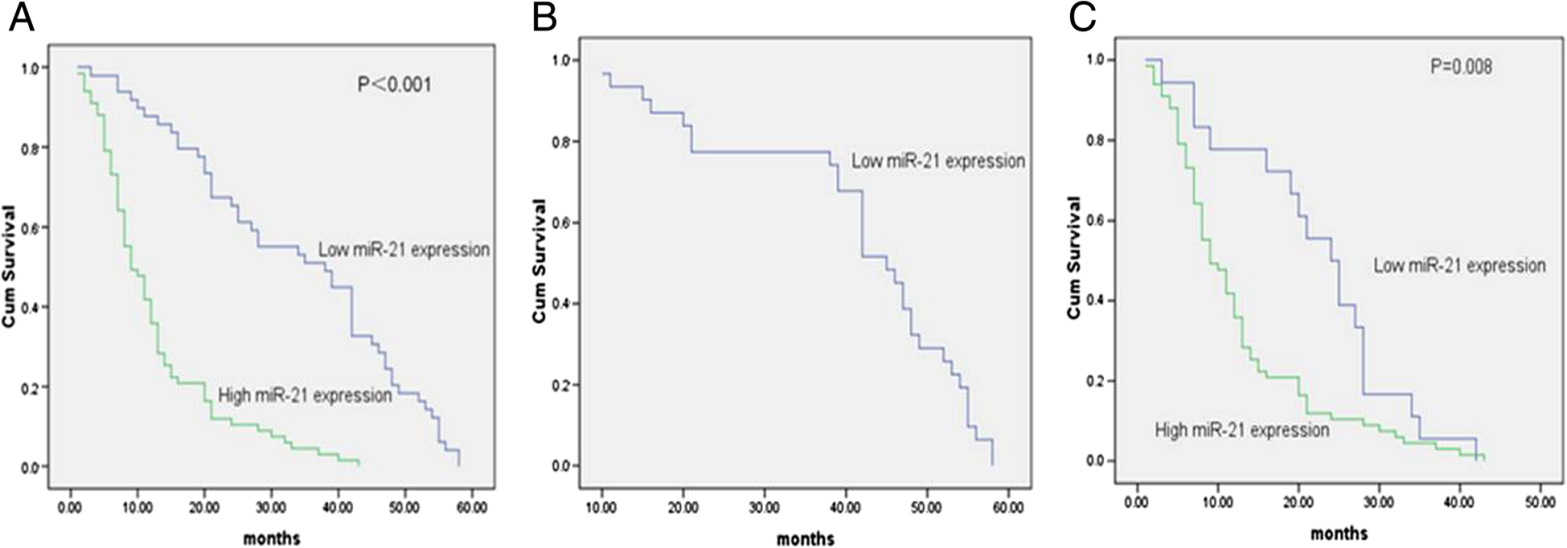
**Kaplan-Meier survival curves for 152 glioma patients with high or low expression of miR-21. (A)** The 5-year overall survival rate of 152 glioma patients with high or low miR-21 expression; **(B)** The 5-year overall survival rate of glioma patients with lower grade (WHO Grades I and II) in high or low miR-21 expression group. **(C)** The 5-year overall survival rate of glioma patients with high grade (WHO Grades III and IV) in high or low miR-21 expression group.

To investigate the relationship between miR-21 expression and clinical outcomes, we reviewed the clinical information for all 152 glioma patients. During the follow-up period, 36 glioma patients (23.68%) were still alive, but 116 patients (76.32%) died (85 from high miR-21 expression group, and 31 from low miR-21 expression group). Kaplan-Meier survival curves suggested that glioma patients with low miR-21 expression had a significantly longer survival time than those with high miR-21 expression (P < 0.001; Figure 2). The survival rate of high miR-21 expression was lower than the survival of other patients, as determined by the log-rank test (p < 0.001). The Cox proportional hazards model was adjusted for several clinical parameters, and these data are shown in Table 3. The Cox proportional hazards model indicated that KPS and WHO grades were associated with overall mortality, but that age, gender, the extent of resection and type of adjuvant treatment were not. Multivariate analysis also showed that high miR-21 expression was a significant and independent indicator of poor prognosis for patients with glioma.

**Table 3:** Cox multivariate analysis

**Parameter**	**Risk**	**95% confidence interval**	***P***
**Age**	1.01	0.996-1.018	0.22
**Gender**	1.12	0.873-2.214	0.56
**WHO grade**	8.17	1.529-5.158	<0.001
**KPS**	0.29	0.204-1.131	<0.001
**MiR-21 expression level**	3.17	2.39-4.179	<0.001

## Discussion

Glioma is one of the most common malignant tumors with a low survival rate, especially in the face of high WHO tumor grades (III, IV). Accurately predicting postoperative outcomes in glioma patients and choosing the correct adjuvant therapy is a present and persistent problem. At this time, traditional pathologic variables are used to make such predictions. Currently, WHO glioma grades are the gold standard for determining glioma patient prognosis, but this information is less useful for individual patients and often fails to discriminate the biological nature of a large number of gliomas. Therefore patients at the same disease stage often have significant discrepancies in survival times. Until now, there are several studies showed that single molecule could be the relapse and prognosis indicator [17,18]. Thus, discovering more molecular markers associated with the relapse and prognosis of glioma is essential.

To date, studies have shown that miRNA expression is correlated with clinical and biological features of tumors, including tissue type, differentiation and aggressiveness, which can be a potential biomarker for therapy and prognosis [10,12-14]. MiR-21 is one of the first discovered miRNAs that is known to be widespread in human tissues. Up-regulation of miR-21 has been observed in numerous human cancers [13,15]. Several publications confirm that miR-21 can promote cell proliferation and invasion and that it inhibits cell apoptosis [19-21]. Inhibition of miR-21 could upregulate the expression of a miRNA-target tumor suppressor gene such as protein tyrosine phosphatase (PTEN) and B cell translocation gene 2 (BTG2), and thereby decreases tumor progression [22-24]. However, to date there has been no research on the prognostic role of miR-21 in glioma. New molecular prognostic factors for glioma may contribute to a better assessment of survival probability and the tailoring of treatments for individual patients.

We investigated miR-21 expression in glioma and determined if this marker could predict disease relapse and patient outcomes. We observed that increased miR-21 expression was evident in human glioma tissues compared with non-neoplastic brain tissues. High miR-21 expression in glioma tissues was significantly correlated with aggressive clinicopathological features as well. Kaplan-Meier analysis revealed that glioma patients with high miR-21 expression had poorer overall survival. Moreover, multivariate analysis revealed that high miR-21 expression was a marker of worse patient outcomes which was independent of known clinical prognostic indicators such as KPS and WHO grades. Gliomas with grade I have different genetic abnormality compared with other grade gliomas. In this study, we divided all the gliomas into two groups: low grade glioma (WHO grade I and II) and high grade glioma (WHO III and IV) and performed multivariate analysis. The result also indicated that miR-21 could be the marker of poor outcomes in these two groups (Relative Risk is 4.947, *P* value < 0.001, 95% confidence interval is 3.12-7.843). All these data indicate that increased expression of miR-21 is correlated with poor glioma patient outcomes. Thus, miR-21 expression may serve as a potential biomarker for overall survival prediction in this patient population.

## Conclusions

Our results revealed that miRNA-21 was overexpressed in glioma tumors and that high expression level of miRNA-21 was strongly associated with poor prognosis, but further prospective studies are needed to determine the actual clinical relevance of this observation. Our data suggest that miR-21 may be an appropriate prognostic marker for evaluating advanced tumors with high pathological grades and that this marker may assist with the appropriate choice of adjuvant chemotherapy as well as serve as a potential therapeutic target in molecular therapy for glioma.

## Competing interests

The authors declare that they have no competing interests.

## Authors’ contributions

ZZ and JZ designed the study. LW participated in the design and coordination, performed the molecular genetic evaluation, and drafted the manuscript. All the patients were followed up by GL and HQ. And DF performed the statistical analysis, and joined into drafting the manuscript. WL, ZZ and JZ all contributed to improving the draft of the manuscript. All authors have read and approved the final manuscript.
